# Copious vaginal discharge finally diagnosed as cervical adenocarcinoma: A case report

**DOI:** 10.1097/MD.0000000000033614

**Published:** 2023-04-21

**Authors:** Peiyu Mao, Chen Zhang, Xinyan Wang, Huadi Yang

**Affiliations:** a Department of Gynecology and Obstetrics, The First Affiliated Hospital of Zhejiang Chinese Medical University, Hangzhou, Zhejiang, China.

**Keywords:** case report, cervical adenocarcinoma, vaginal discharge

## Abstract

Copious vaginal discharge is a frequent manifestation of reproductive tract infections. However, when little effect can obtain treated as vaginitis, cervical disease should be highly suspected.

**Patient concerns:**

A 41-year-old woman had suffering from abnormally increased vaginal discharge without any other signs of discomfort for the past 4 years. A lot of medical examinations and treatment of vaginosis were administered, resulting in unclear diagnosis and little effect.

**Diagnoses:**

Cervical adenocarcinoma.

**Interventions:**

Gynecological examination, vaginal microbiome culture, and primary cervical cancer screening were negative, and a positron emission tomography revealed an increased ^18^F-fluorodeoxyglucose metabolism in the local cervix. After a thorough description, the patient demanded a hysterectomy and bilateral salpingo-oophorectomy.

**Outcomes:**

Histopathological evaluation confirmed adenocarcinoma in situ of the uterine cervix.

**Lessons:**

The correct diagnosis of symptomatic patients with increased vaginal discharge is challenging. Human papillomavirus-negative patients presenting profuse watery vaginal discharge with an abnormal signal of cervix lesion on positron emission tomography or magnetic resonance imaging should be alert to cervical adenocarcinoma. Deep-seated cervical biopsy, conization, or even hysterectomy is conducive to early diagnosis, treatment and improvement of prognosis.

**Key Points**

HPV-negative patients presenting profuse watery vaginal discharge should be alert to cervical adenocarcinoma. Deep-seated cervical biopsy, conization or even hysterectomy is conducive to early diagnosis, treatment and improvement of prognosis.

## 1. Introduction

An abnormal vaginal discharge, as a frequent manifestation of reproductive tract infections, is always considered to be related to vaginosis, including sexually transmitted infections, vulvovaginal candidiasis, and bacterial vaginosis.^[1]^ While, recurrent abnormal vaginal discharge with little effect treated as vaginosis and without much other information to work with, is often a conundrum to clinicians to diagnose and administer treatment. This case describes a women suffered from copious vaginal discharge and confirmed as cervical adenocarcinoma by pathology after hysterectomy.

## 2. Case presentation

A divorced childless 41-year-old woman with bachelor degree had suffering from abnormally increased vaginal discharge, presenting as an annoying and copious, faint yellow vaginal discharge leading to an impression by the patient of smelly, unremittingly having wet underwear for the past 4 years. During the past 4 years, she visited tens of hospitals and a lot of medical examinations and treatment of vaginosis were administered, resulting in unclear diagnosis and little effect. Her menstrual cycle was regular and had no miscarriage ever. She did not have any signs of discomfort such as fever or pelvic pain and denied sexual activity for 6 years after her divorce.

In December 2022, she was tested for human papillomavirus (HPV) and ThinPrep cytology test (TCT) for primary cervical cancer screening and showed negative results in local hospital. An ultrasound examination suggested the possibility of adenomyosis (uterus size of 5.1 * 4.0 * 4.0 cm), endometrial polyps (size of 0.8 * 0.5 cm) and multiple cervical cysts (maximum size of 1.4 * 1.3 cm). At the patient strong request, a hysteroscopic endometrial polypectomy and cervix loop electrosurgical excision procedure was performed, postoperative pathology showed endometrial polyps and low-grade squamous intraepithelial lesion. However, copious vaginal discharge persisted after the surgery.

**Figure 1.**
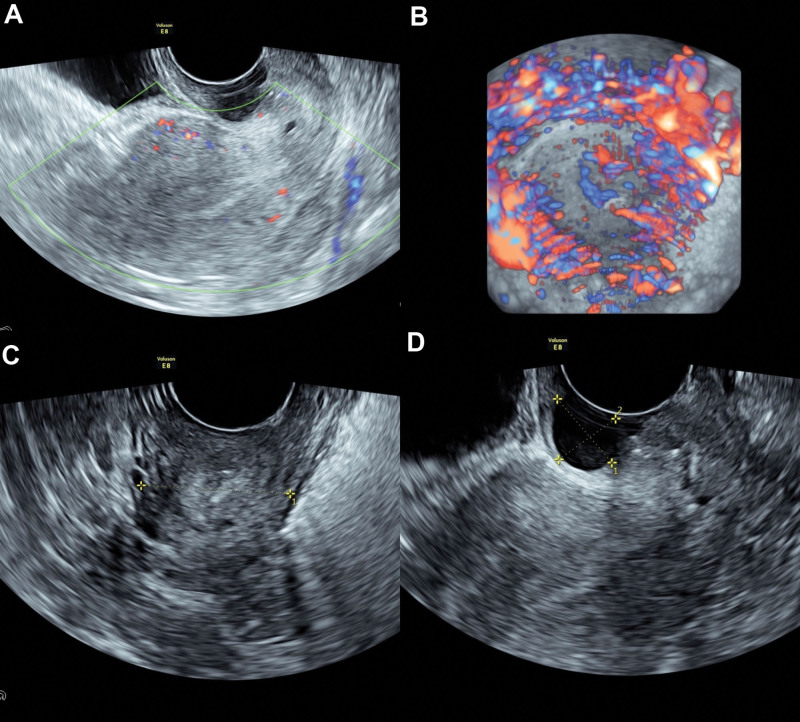
The ultrasound examination suggested a 5.1*4.0*4.4 cm uterus with normal blood supply and endometrium with thickness of 0.7 cm (A); a cervix with uniform blood supply (B) and without space-occupying lesion (C) expect for multiple cervical cysts, whose maximum size was 1.6*1.3 cm (D).

**Figure 2.**
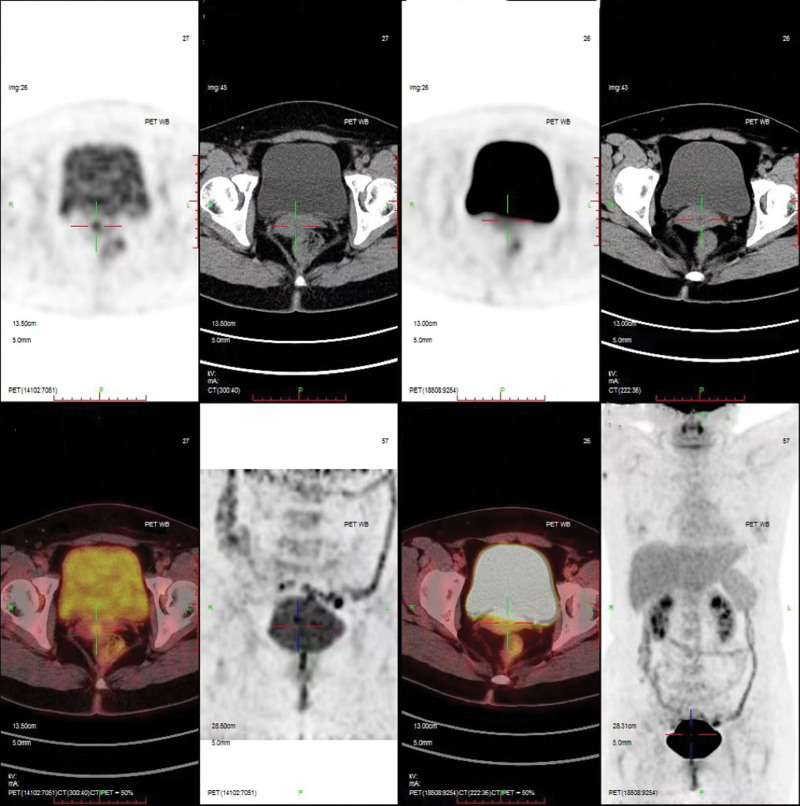
The positron emission tomography (PET) revealed an increased ^18^F-fluorodeoxyglucose (^18^F-FDG) metabolism in the local cervix (line marking).

**Figure 3.**
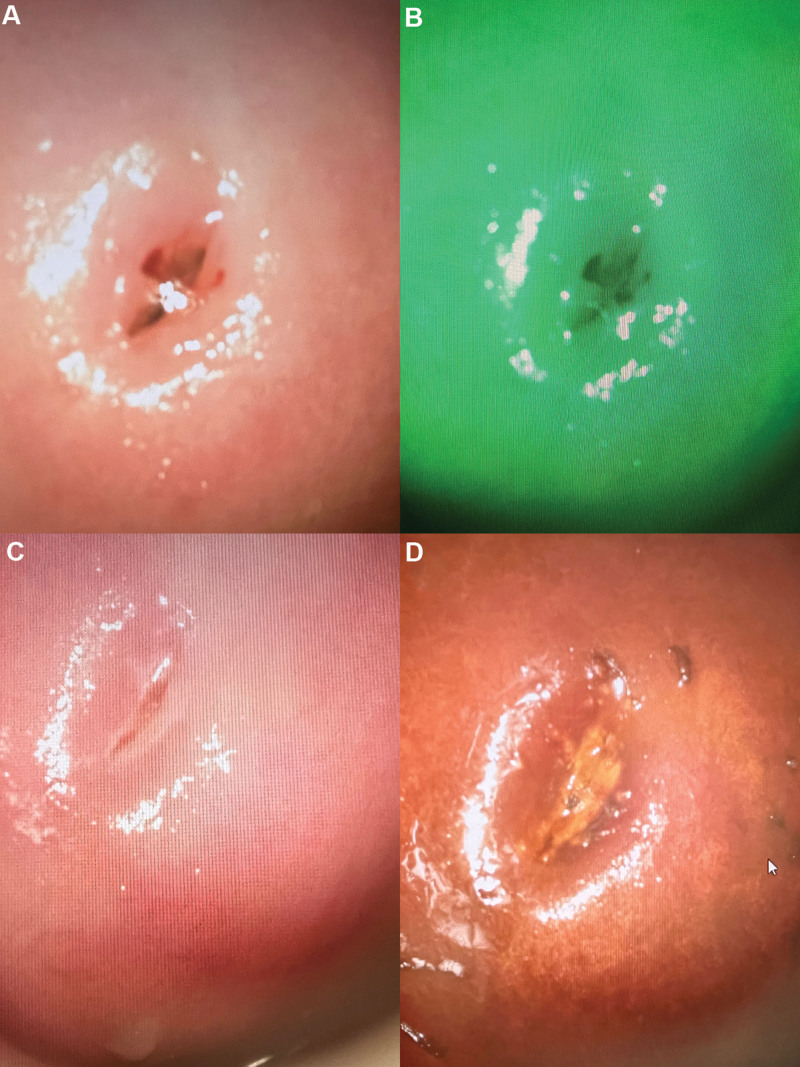
Image representation under colposcopy. No obvious gland openings (A) and no atypical vessels (B) can be observed on the surface of the cervix. The acetowhite epithelium was thin (C) and Lugol staining staine (D).

In January 2023, she was admitted to the gynecological ward in our hospital, with the aim of clear diagnosis and symptoms control. Gynecological examination revealed a mobile uterus of normal size and inaccessible double tubo-ovary without pain. Neither candida, trichomonas, and clue cells; nor chlamydia trachomatis, cervical mycoplasma and gonococcus were detected in vaginal discharge. HPV was negative tested by both hybrid capture II and polymerase chain reaction. TCT was also negative. An ultrasound examination suggested no obvious abnormality (Fig. 1). A positron emission tomography (PET) revealed an increased ^18^F-fluorodeoxyglucose metabolism in the local cervix (Fig. 2). Colposcopy was performed, clear watery secretions covering the cervix can be seen; after wipping off with a cotton swab, no obvious gland openings can be observed on the surface of the cervix. The acetowhite epithelium was thin and Lugol staining stain (Fig. 3). And negative serum tumor markers were detected.

A provisional diagnosis of cervical lesion was made. The patient was given a thorough description and she demanded a hysterectomy and bilateral salpingo-oophorectomy. After she provided written informed consent, a laparoscopy was performed. During the operation, there was no ascites in the pelvic cavity, and normal size uterus, bilateral fallopian tubes and ovaries were seen.

**Figure 4.**
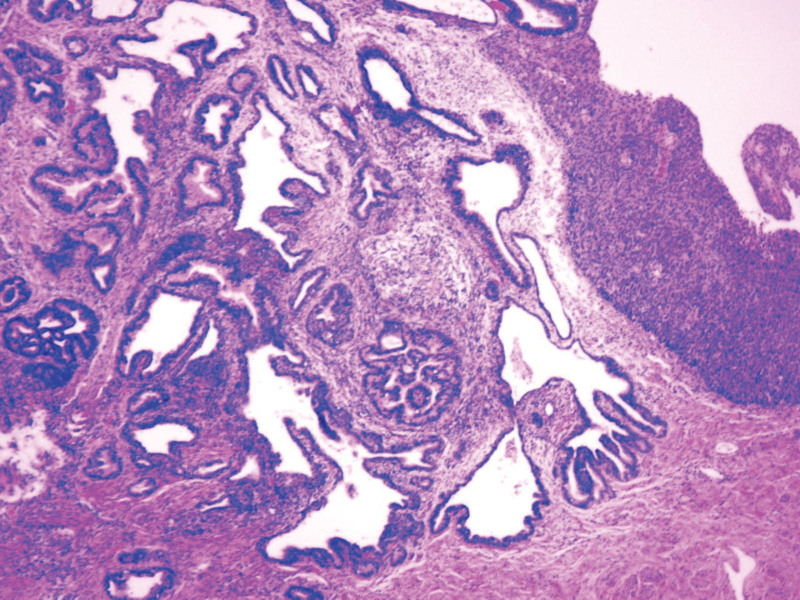
Histopathological evaluation confirmed usual adenocarcinoma in situ of the uterine cervix.

Uterus dissection showed that the cervical tube is cylindrical without any obvious space-occupying lesion, and the local tissue is thickened. There is no abnormality in endometrium and myometrium. Much mucus was found in the uterine cavity and cervical canal. Frozen section pathology suggested cervical dysplasia. Postoperatively, the patient was sent to the ward and recovered well. Histopathological evaluation confirmed usual adenocarcinoma in situ of the uterine cervix (Fig. 4).

## 3. Discussion

Cervical cancer is the leading cause of cancer deaths in women in the developing world,^[2]^ which remains the fourth most common cancer in women worldwide.^[3]^ HPV infection is recognized as a major causative factor in the development of cervical cancer.^[4]^ Primary cervical cancer screening (HPV and TCT) make it a highly preventable disease and can be easily treated if detected at early stages.^[5]^

However, HPV-negative cervical cancer accounts for approximately 3% to 8% of all cases,^[6]^ while cervical adenocarcinoma is frequently (15%–38%) HPV-negative.^[7]^ In this case, both hybrid capture II and polymerase chain reaction were applied to test HPV in order to avoid false-negative results by sampling errors as well as pitfalls in classification accuracy of HPV test. HPV-negative cervical cancer is an easily neglected disease due to lack of interest of clinicians, whom with a misconception to consider HPV infection as the sole cause of cervical cancer. It is reported that the majority of studies about cervical cancer have aimed to discover the viral oncogenic mechanism of HPV and develop diagnostic methods for early detection, HPV vaccines, and targeted drugs for patients with HPV-positive cancer.^[8]^ This might lead to very limited number of studies focus on HPV-negative cervical cancer. Nevertheless, it must be emphasized that patients with HPV-negative cervical cancer had significantly advanced International Federation of Gynecology and Obstetrics stage at diagnosis, lower disease free survival, poorer overall survival and higher risk of progression and mortality than those HPV-positive cervical cancer.^[7]^ Interest in HPV-negative cervical cancer needs to be improved.

Adenocarcinoma currently accounts for 10% to 25%^[9]^ of all uterine cervical carcinomas and has a variety of histopathological subtypes. It classically arises in middle-aged women with symptoms, including profuse watery vaginal discharge and abnormal uterine bleeding.^[10]^ When profuse vaginal discharge cannot be relieved after treatment of vaginitis, further examination in differential diagnosis of cervical adenocarcinoma is needed.

Cervical tumor can be evaluated by several imaging modalities, and it has been reported that squamous carcinoma of the cervix (SCC) tended to be hypoechoic and adenocarcinoma tended to be isoechoic on ultrasound.^[11]^ Computed tomography is limited by poor soft tissue contrast, and magnetic resonance imaging is superior to CT for local evaluation.^[12]^ Though there was no significant difference in standardized uptake value max between SCC and non-SCC on 18F-fluorodeoxyglucose PET,^[13]^ an increased metabolism of cervix is a signal of cervix lesion.

Patients suffering from large amount of vaginal discharge had cervical lesions on magnetic resonance imaging/PET showing abnormal signal should be alert to cervical adenocarcinoma. Deep-seated cervical biopsy and even conization is conducive to early diagnosis, treatment and improvement of prognosis. Hysterectomy is an optional method for patients without fertility requirements whose symptoms persist constantly for a long time.

## Author contribution

**Conceptualization:** Xinyan Wang.

**Data curation:** Peiyu Mao, Chen Zhang.

**Resources:** Peiyu Mao.

**Supervision:** Peiyu Mao, Xinyan Wang, Huadi Yang.

**Validation:** Peiyu Mao.

**Visualization:** Peiyu Mao.

**Writing – original draft:** Peiyu Mao.

**Writing – review & editing:** Peiyu Mao, Huadi Yang.
